# Perceived enjoyment from virtual experience to real-world care: how Chinese digital games containing TCM culture influence players’ TCM treatment intention

**DOI:** 10.3389/fpubh.2026.1827198

**Published:** 2026-06-02

**Authors:** Jinxiao Ding, Yuxin Jiao, Lin Ji

**Affiliations:** 1 Artificial Intelligence and Big Data College, Chongqing Polytechnic University of Electronic Technology, Chongqing, China; 2 School of Agricultural Economics and Rural Development, Renmin University of China, Beijing, China

**Keywords:** digital games, gaming motivation, health self-efficacy, TCM cultural identity, TCM treatment intention

## Abstract

**Background:**

In the context of *Health China 2030*, the Chinese government endorses the unique advantages of traditional Chinese medicine (TCM) within the public health care system and promotes the inheritance and development of TCM culture. Emerging digital formats—such as online literature, online audiovisual media, and digital games—have become important vehicles for promoting TCM culture and public health education. Consequently, Chinese digital games have increasingly incorporated elements of TCM, serving as new media for communicating health knowledge and cultural values to a broad player population. This study examines how exposure to digital games containing TCM culture, supported by national health promotion policies, influences players’ willingness to accept TCM treatment, thereby contributing to the broader goal of improving residents’ health welfare. However, empirical research examining how such games influence players’ willingness to accept TCM-based medical treatment remains limited. This study aims to investigate the key factors and mechanisms through which digital game use motivations shape players’ intention to seek TCM treatment.

**Methods:**

Based on data collected through an online questionnaire survey of 460 Chinese digital game players, PLS-SEM was utilized to examine the core factors and relationships between gaming motivations, TCM cultural identity, health self-efficacy, and TCM treatment intention.

**Results:**

The findings reveal that three types of game use motivations—hedonic, social, and knowledge-seeking—indirectly enhance players’ willingness to accept TCM treatment by strengthening their health self-efficacy and identification with TCM culture. Additionally, players’ distrust of the TCM healthcare system negatively moderates the association between gaming motivations and TCM treatment intention.

**Conclusion:**

This study demonstrates the potential of digital games as effective tools for integrating cultural transmission with public health communication. By improving players’ TCM treatment intention, digital games containing TCM culture contribute to the public health objectives outlined in the Health China 2030 strategy. The results provide both theoretical grounding and practical guidance for government agencies and enterprises seeking to leverage digital games to promote TCM cultural inheritance and enhance public health education as part of health welfare improvements.

## 1 Introduction

*The Health China 2030*, a cornerstone of China’s public health care system, explicitly mandates the integration of TCM with Western medicine to improve residents’ health welfare. This national policy emphasizes that the public should acquire and master basic TCM health literacy, and urges medical institutions to vigorously promote and apply appropriate TCM technologies and methods. The 14th Five-Year Plan for the Development of Traditional Chinese Medicine explicitly stipulates promoting the integrated development of traditional Chinese medicine with animation and games. As part of this policy framework, emerging digital formats—including digital games containing TCM cultural elements—have been recognized as innovative tools for public health education and cultural transmission. These games not only disseminate TCM knowledge but also potentially contribute to players’ health literacy, thereby aligning with the public health care system’s goal of enhancing population health outcomes.

Traditional Chinese Medicine (TCM) is not merely a subset of complementary and alternative medicine (CAM); rather, it has profound historical roots and an independent theoretical framework, and plays a pivotal role in the public health care system. Nonetheless, due to reasons such as public doubts about the professionalism of the TCM health care system, public treatment intention toward TCM faces significant challenges in practice, as it is often not the primary choice for individuals seeking medical help. Furthermore, despite the top-down promotion through Health China 2030 and the 14th Five-Year Plan for the Development of Traditional Chinese Medicine, the translation of TCM knowledge into improved health outcomes and treatment intention among the public remains a complex challenge. Therefore, it is essential to enhance the dissemination of TCM knowledge and culture, so as to foster greater public recognition of its value and professionalism, and ultimately promote public intention to seek TCM treatment while simultaneously improving players’ health self-efficacy and TCM cultural identity, key indicators of health welfare targeted by the public health care system.

Existing research on treatment intention has mainly explored influencing factors from the perspectives of patients’ demographics and clinic factors, medicine distrust, public cultural identity, and individuals’ self-efficacy. Specifically, patients’ demographic characteristics such as region, age, educational level, and gender, as well as clinical characteristics including disease type and disease severity (1, 2), influence TCM treatment intention. Medicine trust (such as trust in physicians’ professional expertise, medical information transparency, trust in authorities’ medical security system, and trust in medical systems of non-ethnic groups) also affects treatment intention (3–10). Cultural identity, such as identity of local traditional culture, values of national culture, and exclusivity toward other ethnic groups, also influences treatment intention. Individuals’ self-efficacy influencing treatment intention encompasses confidence in one’s own health management, the ability to understand relevant health knowledge, and perceptions of similar health behaviors among others (11–13). Nevertheless, the existing research has rarely examined how digital media interventions, supported by national health policies, influence health-seeking behaviors within the context of China’s social security system. First, limited scholarly work has investigated the mechanisms of emerging digital media, such as digital games, in TCM health communication and health education. Most studies focus on why patients choose TCM, without considering the impacts of the public’s motivation on TCM treatment intention under non-specific disease conditions. Second, when analyzing the internal psychological pathways through which motivation translates into actual intention, existing research lacks a systematic introduction of mediating variables. Few studies have integrated TCM cultural identity and health self-efficacy into a unified framework to explore their joint mediating role in enhancing treatment intention, which limits the theoretical explanatory power of how motivation is converted into behavior and contributes to health welfare. Finally, most existing studies focus on positive facilitating factors, and have not sufficiently explored how distrust in the TCM healthcare system, as an institutional negative moderator, exerts negative effects on the motivation–behavior pathway within the complex digital media environment.

To narrow the gap, this study situates itself within the context of digital communication and a series of policy frameworks concerning TCM, and examines the influencing mechanism of the motivation to engage with digital games containing TCM culture on TCM treatment intention. Specifically, this study classifies the motivational factors for participating in TCM-themed games into hedonic motivation, social motivation, and knowledge-seeking motivation, and introduces TCM cultural identity and health self-efficacy as mediating variables. Moreover, this study examined the mediating role of TCM health care system distrust in the relationships between gaming motivation and TCM treatment intention. This study supplemented the empirical analysis of PLS-SEM based on data from 460 game players.

This study offers three potential contributions to TCM treatment intention. First, this study broadens the research perspective of TCM treatment intention and public health care research. Most prior research focuses on the adoption intention of traditional Chinese medicine among patients with specific diseases (2), whereas this study focuses on players’ motivation to use digital games containing TCM culture and analyzes the effects of hedonic motivation, social motivation, and knowledge-seeking motivation on TCM treatment intention among the general public. This enriches the research on motivation and effects in TCM health communication, reveals how the public’s active digital use can be transformed into potential treatment intention, and demonstrates that the effects of health communication are influenced by media forms and evolve with changes in interactive media formats within the broader context of social public health policy. Second, the mediating mechanisms of TCM cultural identity and health self-efficacy were further explored, addressing the research gap of missing potential mediating effects in TCM treatment intention. Existing studies tend to describe the direct impacts of external factors on treatment intention (14, 15), with little consideration of the psychologically stimulating effect of cultural identity on the pathway from motivation to intention. Third, this study examines the moderating role of distrust of the TCM health care system in the motivation–intention relationship. This may provide a new explanation for why strong motivation and high-quality digital games do not always effectively promote the public’s health-seeking behavior in practice. By introducing this moderating variable, this study responds to discussions in the existing literature on the complex relationship between medicine distrust and treatment intention (16, 17).

## 2 Hypothesis development

### 2.1 Gaming motivation and TCM treatment intention

According to the uses and gratifications theory, individuals actively select specific media based on their own needs and obtain corresponding gratifications by acquiring information or facilitating specific behaviors (18). When players satisfy their emotional, social, and cognitive needs through games, this will translate into recognition and action toward game-related TCM treatment. First, hedonic motivation refers to the motivation for players to play games in order to experience pleasure and fun (19). Sheng et al. found that hedonic motivation is an important factor in improving the level of physical health activity among college students (20). Previous studies on player behaviors regarding digital games or serious games have pointed out that acquiring health knowledge and changing health behaviors occur simultaneously during the process of entertainment (21). When players obtain pleasure in games, they will also develop relevant treatment intention through social modeling effects (22). Some studies have acknowledged that hedonic motivation influences continued intention (23). Drawing on the preceding discussion, one may plausibly deduce that players’ confirmation of hedonic motivation serves to elevate users’ help-seeking intention (HSI).

Second, social motivation can be defined as the manner in which people participate in games to connect and interact with peers (24). The interaction among players acts as a key factor in the design and running of digital games, exerting a notable impact on players’ gaming experiences, engagement degrees, and understandings of TCM (25). Consequently, previous research shows that social motivation enhances players’ intention.

Third, previous studies have noted that knowledge-seeking motivation is an important driver for people to use new media such as social media and digital games. In virtual communities, players’ perceived acquisition and improvement of relevant knowledge, as well as the credibility of such knowledge, are key factors influencing knowledge-seeking motivation (25). Players are likely to change their behavior when they obtain knowledge from social media (26). When players perceive knowledge growth, it results in positive attitudes toward behavior intention. Therefore, the following hypotheses are proposed:

*H1*. Hedonic motivation (HM) positively affects HSI.

*H2*. Social motivation (SM) positively affects HSI.

*H3*. Knowledge-seeking motivation (KSM) positively affects HSI.

### 2.2 The mediating role of TCM cultural identity and health self-efficacy

Cultural identity broadly denotes an individual’s sense of belonging to and identification with a particular cultural group, such as an ethnic group or a nation. By comparison, TCM cultural identity is a domain-specific cultural identification that reflects individuals’ emotional attachment, value recognition, and behavioral inclination toward traditional Chinese medicine culture. According to social identity theory, when digital media satisfies audiences’ social and emotional needs through strategies such as storytelling and social identity construction, audiences may develop a psychological attachment to the value system conveyed (27). Game-related research further indicates that shaping player identity can substantially enrich users’ hedonic gaming experience (28). In the context of this study, digital games containing interesting interactive mechanisms and plot designs, together with sharing and communication in game communities, enable players to immerse themselves in TCM culture imperceptibly and integrate TCM philosophy into their personal identity. Furthermore, this may lead individuals to prefer TCM treatment that is consistent with their cultural identity when seeking medical care or conducting self-health management.

Accordingly, this study proposes the following hypothesis:

*H4a*. CI positively mediates the relationship between HM and HSI.

*H4b*. CI positively mediates the relationship between SM and HSI.

*H4c*. CI positively mediates the relationship between KSM and HSI.

Distinct from TCM cultural identity, health self-efficacy (HSE) originates from social cognitive theory and reflects individuals’ perceived confidence in their capability to adopt and manage TCM health behaviors; it is a capability-based psychological mechanism, whereas TCM cultural identity is a value-based and belonging-based mechanism. The two represent independent psychological pathways linking gaming motivation to health-seeking intention. Previous studies have shown that self-efficacy is individuals’ confidence in their ability to perform specific behaviors. It can directly affect people’s understanding of health-related knowledge (29) and further positively affect treatment intention. A pleasant gaming experience can lower the psychological barrier for players to understand TCM knowledge. Discussions on TCM knowledge among players help strengthen individuals’ health efficacy expectations. Meanwhile, self-efficacy is an indirect factor related to health behaviors (30). If players learn or master TCM knowledge through games but lack the confidence or ability to manage their own health, it will still be difficult to transform into a treatment intention. Accordingly, this study proposes the following hypothesis:

*H5a*. HSE positively mediates the relationship between HM and HSI.

*H5b*. HSE positively mediates the relationship between SM and HSI.

*H5c*. HSE positively mediates the relationship between KSM and HSI.

### 2.3 TCM health care system distrust as a moderator

Distrust in the TCM healthcare system refers to an individual’s skeptical perception toward institutional deficiencies, profit-driven dishonesty, and adverse experiences within the TCM healthcare system. According to social cognitive theory, individuals’ distrust in the TCM healthcare system weakens their positive outcome expectations of TCM therapies, thereby inhibiting their behavioral intention to seek TCM medical services. Therefore, the influence of gaming motivation on TCM treatment intention may be moderated by TCM health care system distrust (HSD). The direct evidence was not found in previous studies regarding the moderating effect of TCM health care system distrust on gaming motivation and treatment intention. However, medical distrust is one of the main barriers to public health-seeking behavior (31). Although players may gain enjoyment, social interaction, and knowledge growth through digital games containing TCM culture, these satisfactions mainly occur at the level of virtual interaction. When players distrust the health care system, such virtual interactions can hardly be effectively converted into treatment intention (32, 33). Therefore, this study hypothesizes:

*H6a*. TCM HSD negatively moderates the relationship between HM and HSI.

*H6b*. TCM HSD negatively moderates the relationship between SM and HSI.

*H6c*. TCM HSD negatively moderates the relationship between KSM and HSI.

**Figure 1:**
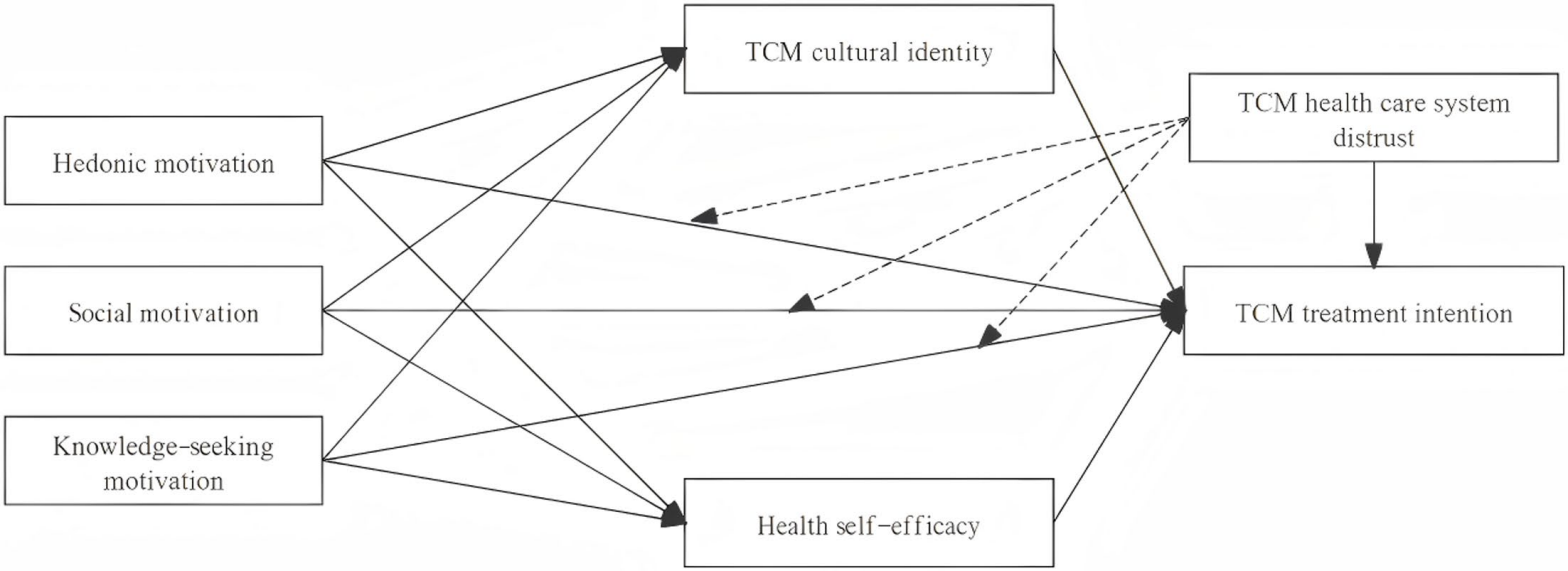
The research framework.

Given the above analysis, this study constructed the framework depicted in Figure 1.

## 3 Data and methodology

### 3.1 Sample and data collection

Data for this study were collected online in January 2025, with respondents’ experience of playing Chinese digital games containing TCM culture. The decision to recruit players as respondents was guided by the considerations detailed hereafter. First, considering factors such as the geographical distribution and time flexibility of game players in the data collection process, an online questionnaire survey was the most accessible approach to reach this specific research population. Second, respondents were required to have played at least one game containing TCM cultural elements, with a minimum playtime of more than 1 h, taking into account the “flow” experience designed in most games. Finally, players of different age groups vary in their attitudes, cognition, and behaviors toward TCM, as well as in their health concerns. An online survey was able to cover players across different age groups to ensure the representativeness of the sample. To identify respondents exposed to TCM-containing games, we included a screening question at the beginning of the questionnaire: “Have you ever played digital games containing TCM cultural elements for no less than 1 h?” Respondents who answered “Yes” were regarded as eligible participants.

This study distributed the questionnaire link through QQ and WeChat and recruited 22 participants to undertake the pilot survey. These 22 respondents included 15 game players, 3 TCM doctors, 2 game research scholars, and 2 product managers from game companies. Through the pre-survey, the respondents examined and provided revisions on the logical consistency, item expression, and relevance of the questionnaire. Drawing on responses gathered throughout the pre-survey, this study revised ambiguous, logically inconsistent, and hard-to-understand parts of the measurement items to ensure the questionnaire was comprehensible to respondents. During the formal research phase, Wenjuanxing^1^ was adopted as the dedicated platform to distribute research questionnaires, and data were collected via convenience sampling. To ensure the quality of the questionnaire, this study required respondents to participate using a unique IP address, prohibited duplicate submissions, and restricted the response time to between 7 and 15 min. Based on response time and IP address uniqueness, we excluded 28 invalid questionnaires. Questionnaires with a large number of missing values were excluded. Finally, after filtering out invalid questionnaires, a total of 460 valid responses were obtained.

The sample profiles are presented in Table 1. The proportion of male gamers was slightly higher than that of female gamers, with the former accounting for 54.68% and the latter for 45.32%. Nearly 86% of respondents were aged 21–40, and 78% were married. More than 80% of the respondents possessed a bachelor’s degree. In addition, 64.49% of respondents played digital games 2–3 h per day, who are so-called hardcore gamers. Nearly 50% of respondents had 8 years or more of gaming experience.

**Table 1:** Demographic profile of respondents.

Characteristic	Demographic	Frequency	Percentage
Gender	Male	251	54.68%
	Female	209	45.32%
Age (years)	20 and below	8	1.74%
	21–30	148	32.24%
	31–40	246	53.38%
	41–50	40	8.71%
	51 and above	18	3.92%
Educational	Senior high school or below	7	1.53%
	Associate degree	36	7.84%
	Bachelor’s degree	368	80.17%
	Master’s degree and above	49	10.46%
Marital status	Married	357	77.78%
	Unmarried	103	22.22%
Gaming time (daily)	1 h per day	123	26.80%
	2–3 h per day	297	64.49%
	More than 4 h per day	40	8.71%
Gaming experience (years)	1 year and below	6	1.31%
	3–5 years	97	21.13%
	6–7 years	137	29.85%
	8 years and above	220	47.71%
Total		460	100

Source(s): Authors’ own work.

### 3.2 Measures

The questionnaire comprised three sections. The first part of the questionnaire served to obtain respondents’ demographic information. The second section measured their gaming motivation and identity toward TCM culture. The third section assessed their health self-efficacy, TCM health care system distrust, and TCM treatment intention. The measurement items employed in this research were adopted from established original scales and adjusted appropriately. Items measuring hedonic motivation and cultural identity were adapted from the scale developed by Wang and Men (34). Meanwhile, items for hedonic motivation were also referenced from the scale designed by Abbasi et al. (35). Items measuring social motivation were derived from the social motivation scale for online game players established by Yee (24), who categorized social motivation into three dimensions: Socializing, Relationship, and Teamwork. Hou (36) and Dalisay et al. (37) further refined the measurement of social motivation based on this framework, and this study conducted context-specific adaptations accordingly. Knowledge-seeking motivation was adapted from the Seeker knowledge growth scale developed by He and Wei (38). The TCM health care system distrust was adapted from the scale developed by Armstrong et al. (33) and revised by Shea et al. (39). Measurement of TCM treatment intention was adapted from the Help-Seeking Intention Scale developed by Romero Reyes et al. (40).

Since all original scales were derived from foreign literature, we strictly followed the standard forward-backward translation procedure. First, two researchers independently translated the English scales into Chinese. The translated items were then reviewed and refined through expert panel discussion to ensure content validity and cultural applicability, resulting in the final questionnaire. Subsequently, another researcher back-translated the Chinese version into English. Any semantic discrepancies were resolved through group discussion to achieve linguistic and semantic equivalence between the original and adapted scales. A five-point Likert scale ranging from “1” for “strongly disagree” to “5” for “strongly agree” was employed to assess the items of all constructs.

## 4 Data analysis and results

### 4.1 Common method variance

This study conducted a common method variance (CMV) test using Harman’s single-factor test (41). Results indicated that 41.77% of the total variance in the factor analysis was explained by the first factor, which was much less than the 50% threshold (42). In addition, Table 2 shows that the Variance Inflation Factor (VIF) values for all items range from 1.436 to 2.311, which are well below the critical value of 5, indicating that there is no serious multicollinearity. These results suggest that CMV does not pose a threat.

**Table 2:** Reliability and validity tests of the constructs.

Construct	VIF	Items	Standard loadings	Cronbach’s α	CR	AVE
HM	2.311	Using Chinese digital games containing TCM culture is more fun than I initially expected	0.892	0.849	0.908	0.768
	1.884	Actually, the process of using Chinese digital games containing TCM culture is more pleasant than I initially expected	0.861			
	2.111	Learning TCM knowledge by Chinese digital games containing TCM culture is more enjoyable than I initially expected	0.876			
SM	1.664	I often have conversations with other players about TCM while playing games	0.825	0.805	0.882	0.713
	1.719	It’s important for me to discuss with other players about TCM during the game	0.843			
	1.745	I really cherish the chance to talk about TCM with other players while playing games	0.865			
KSM	1.901	Playing digital games containing TCM culture promotes my TCM knowledge growth and development	0.861	0.824	0.895	0.739
	1.807	Playing digital games containing TCM culture reinforces my TCM competence	0.856			
	1.867	Playing digital games containing TCM culture helps me strengthen my concepts in TCM	0.863			
CI	1.570	I have a strong sense of identification with the TCM culture presented in the game	0.837	0.753	0.858	0.668
	1.436	I have an inexplicable sense of intimacy with the TCM culture presented in the game	0.791			
	1.530	I have a strong sense of belonging to the TCM culture presented in the game	0.823			
HSE	1.678	I am confident that I could deal efficiently with my own health	0.846	0.788	0.875	0.701
	1.635	I have the ability to deal with my health	0.826			
	1.621	When I am confronted with a health problem, I can usually find several solutions	0.838			
TCM HSD	1.51	I believe that the TCM health care system puts making money above patients’ needs	0.827	0.751	0.857	0.667
	1.531	I believe that the TCM health care system lies to patients for profit	0.821			
	1.466	I believe that the TCM health care system often leads to medical malpractice	0.802			
HSI	2.056	I want to seek the TCM treatment if I encounter health problems	0.882	0.821	0.893	0.736
	1.758	When I go to see a doctor, I will plan to actively consider TCM treatment	0.845			
	1.787	I intend to use TCM for health preservation and body regulation as part of my future health routine	0.846			

Source(s): Authors’ own work.

(1) CR is short for Composite Reliability; (2) AVE is short for Average Variance Extracted.

### 4.2 The measurement model

First, the reliability of the measurement model was assessed using Cronbach’s alpha and composite reliability (CR). As shown in Table 2, Cronbach’s α coefficients were all greater than 0.75, and composite reliability (CR) values all exceeded 0.85. Both indices are higher than the recommended threshold of 0.7 (43), which indicates that the scale has good internal consistency and composite reliability.

Second, the factor loadings and average variance extracted (AVE) for all items were used to assess the convergent validity (44). The results in Table 2 show that the standardized loadings of all items range from 0.791 to 0.892, exceeding the threshold of 0.7. Additionally, the AVE values for all constructs range from 0.667 to 0.768, all above the 0.5 benchmark, indicating sufficient convergent validity.

Third, we assessed the discriminant validity of the model using three complementary methods. As shown in Table 3, the cross-loading results indicate that each item loads substantially higher on its own latent construct than on other constructs. Table 4 presents the Fornell-Larcker criterion results, with the square roots of the constructs’ AVEs ranging from 0.816 to 0.876 and inter-construct correlations ranging from 0.432 to 0.716. Each construct’s AVE square root exceeds its correlations with all other constructs, satisfying the standard for discriminant validity (45). Table 5 reports the heterotrait-monotrait (HTMT) ratios, for which all construct combinations fall below the conservative threshold of 0.9 (46, 47), and no confidence intervals include 1. Collectively, these three methods validate discriminant validity at complementary analytical levels: cross-loadings provide item-level diagnostics, the Fornell-Larcker criterion offers a variance-based construct-level assessment, and HTMT delivers a stringent inferential test. All three approaches consistently confirm that every construct in this study possesses satisfactory discriminant validity.

**Table 3:** Cross-loadings of the constructs.

Construct	Items	HM	SM	KSM	CI	HSE	TCM HSD	HSI
HM	HM1	0.892	0.540	0.569	0.427	0.487	0.390	0.577
	HM2	0.861	0.486	0.575	0.446	0.533	0.378	0.549
	HM3	0.876	0.477	0.556	0.454	0.486	0.366	0.560
SM	SM1	0.417	0.825	0.479	0.436	0.408	0.426	0.500
	SM2	0.503	0.843	0.478	0.424	0.459	0.456	0.544
	SM3	0.522	0.865	0.476	0.516	0.482	0.488	0.595
KSM	KSM1	0.560	0.450	0.861	0.447	0.478	0.362	0.579
	KSM2	0.575	0.531	0.856	0.454	0.499	0.399	0.607
	KSM3	0.535	0.475	0.863	0.471	0.530	0.392	0.570
CI	CI1	0.417	0.457	0.448	0.837	0.520	0.513	0.592
	CI2	0.376	0.461	0.421	0.791	0.467	0.399	0.517
	CI3	0.444	0.421	0.435	0.823	0.492	0.501	0.575
HSE	HSE1	0.480	0.494	0.516	0.52	0.846	0.482	0.600
	HSE2	0.496	0.411	0.456	0.46	0.826	0.422	0.561
	HSE3	0.465	0.434	0.494	0.533	0.838	0.454	0.633
TCM HSD	TCM HSD1	0.332	0.417	0.402	0.519	0.462	0.827	0.538
	TCM HSD2	0.353	0.465	0.349	0.449	0.463	0.821	0.511
	TCM HSD3	0.375	0.448	0.342	0.446	0.400	0.802	0.499
HSI	HSI1	0.587	0.583	0.611	0.575	0.626	0.560	0.882
	HSI2	0.538	0.548	0.583	0.610	0.623	0.503	0.845
	HSI3	0.524	0.540	0.559	0.587	0.593	0.565	0.846

Source(s): Authors’ own work.

**Table 4:** Correlations and square roots of AVEs (Fornell-Larcker criterion).

Construct	HM	HSE	KSM	SM	CI	TCM HSD	HSI
HM	0.876						
HSE	0.573	0.837					
KSM	0.647	0.585	0.860				
SM	0.572	0.534	0.565	0.844			
CI	0.505	0.604	0.532	0.546	0.817		
TCM HSD	0.432	0.542	0.447	0.542	0.578	0.816	
HSI	0.641	0.716	0.681	0.650	0.688	0.632	0.858

Source(s): Authors’ own work.

**Table 5:** Heterotrait-Monotrait ratio (HTMT) and confidence interval.

Construct	HM	SM	KSM	CI	HSE	TCM HSD
SM	0.691 [0.606, 0.768]					
KSM	0.774 [0.701, 0.837]	0.696 [0.607, 0.778]				
CI	0.632 [0.527, 0.722]	0.702 [0.603, 0.791]	0.676 [0.571, 0.766]			
HSE	0.702 [0.590, 0.801]	0.670 [0.542, 0.787]	0.725 [0.617, 0.830]	0.783 [0.665, 0.897]		
HSD	0.542 [0.412, 0.656]	0.699 [0.596, 0.793]	0.567 [0.445, 0.674]	0.766 [0.630, 0.884]	0.703 [0.571, 0.824]	
HSI	0.768 [0.690, 0.833]	0.799 [0.729, 0.862]	0.828 [0.757, 0.888]	0.876 [0.788, 0.951]	0.890 [0.777, 0.996]	0.806 [0.687, 0.903]

Source(s): Authors’ own work.

HM = hedonic motivation; SM = social motivation; KSM = knowledge-seeking motivation; CI = TCM cultural identity; HSE = health self-efficacy; HSD = TCM health care system distrust; HSI = TCM treatment intention. The values in brackets represent the 95% bootstrap confidence intervals for HTMT.

### 4.3 Path relationship evaluations

This study employs a structural model to assess the impact of gaming motivation on intention to use TCM. The structural model and corresponding research hypotheses are examined using the standard Bootstrapping procedure in Smart PLS 4.0, with 460 samples subjected to 5,000 iterations. The results of the structural model, including control variables, are presented in Table 6.

**Table 6:** The results of the direct effect.

Hypotheses and paths	β	T-value	Confidence intervals
HM → HSE	0.256***	5.096	[0.156, 0.349]
HM → CI	0.167**	3.173	[0.061, 0.265]
HM → HSI	0.118**	3.175	[0.043, 0.190]
HSE → HSI	0.244***	4.556	[0.152, 0.363]
KSM → HSE	0.294***	5.936	[0.197, 0.391]
KSM → CI	0.249***	4.596	[0.143, 0.356]
KSM → HSI	0.203***	5.491	[0.130, 0.275]
SM → HSE	0.222***	4.919	[0.136, 0.315]
SM → CI	0.309***	5.982	[0.208, 0.412]
SM → HSI	0.136**	2.732	[0.035, 0.230]
CI → HSI	0.202***	3.812	[0.098, 0.303]

*p < 0.05, **p < 0.01, ***p < 0.001. HM = hedonic motivation; SM = social motivation; KSM = knowledge-seeking motivation; CI = TCM cultural identity; HSE = health self-efficacy; HSD = TCM health care system distrust; HSI = TCM treatment intention.

Source(s): Authors’ own work.

As anticipated, HM (β = 0.118, *p* < 0.01), SM (β = 0.136, *p* < 0.01), and KSM (β = 0.203, *p* < 0.001) all had positive effects on HSI, validating H1, H2, and H3. Furthermore, all the motivation had positive impacts on CI and HSE. HM positively affected CI and HSE, with standardized regression coefficients of 0.167 (*p* < 0.01) and 0.256 (*p* < 0.001), respectively. SM positively affected CI and HSE, with standardized regression coefficients of 0.309 (*p* < 0.001) and 0.222 (p < 0.001), respectively. KSM positively affected CI and HSE, with standardized regression coefficients of 0.249 (*p* < 0.001) and 0.94 (*p* < 0.001), respectively. In addition, CI (β = 0.202, *p* < 0.001) and HSE (β = 0.244, *p* < 0.001) all exhibited positive relationships with HSI, supporting H4 and H5.

### 4.4 The mediating effects of TCM cultural identification and health self-efficacy

Table 7 presents the results of mediating effect tests, conducted via bootstrapping. The significance of indirect effects was assessed using 95% confidence intervals and T-values (48). Therefore, CI and HSE are significant mediators between HM, SM, KSM, and HSI. Furthermore, the specific mediating effects of HSE (β = 0.062, *p* < 0.001) regarding the relationship between HM and HSI were greater than CI (β = 0.034, *p* < 0.01). Similarly, for the link between KSM and HSI, the specific mediating effects of HSE (β = 0.071, *p* < 0.001) exceeded those of CI (β = 0.050, *p* < 0.01). In contrast, regarding the relationship between SM and HSI, the specific mediating effects of CI (β = 0.062, *p* < 0.01) were slightly greater than those of HSE (β = 0.054, *p* < 0.01). Therefore, the results also confirm H4 and H5. Additionally, the total indirect effects of HM on HSI (β = 0.096, *p* < 0.001) are noticeably lower than those of SM (β = 0.117, *p* < 0.001) and KSM (β = 0.122, *p* < 0.001).

**Table 7:** The results of the mediating effect.

Hypotheses and paths	Specific indirect effects			Total indirect effects			Direct effects			Total effects		
	β	*T*-value	Confidence intervals	β	*T*-value	Confidence intervals	β	*T*-value	Confidence intervals	β	*T*-value	Confidence intervals
HM → CI → HSI	0.034*	2.450	[0.010, 0.063]	0.096***	3.986	[0.053, 0.147]	0.118**	3.175	[0.043, 0.190]	0.214***	5.474	[0.137, 0.289]
HM → HSE → HSI	0.062**	3.081	[0.029, 0.109]									
SM → CI → HSI	0.062**	2.850	[0.025, 0.109]	0.117***	4.380	[0.070, 0.174]	0.136**	2.732	[0.035, 0.230]	0.253***	5.714	[0.164, 0.337]
SM → HSE → HSI	0.054**	2.871	[0.025, 0.099]									
KSM → CI → HSI	0.050**	2.835	[0.020, 0.089]	0.122***	4.890	[0.077, 0.176]	0.203***	5.491	[0.130, 0.275]	0.325***	8.220	[0.248, 0.404]
KSM → HSE → HSI	0.071***	3.504	[0.038, 0.119]									

*p < 0.05, **p < 0.01, ***p < 0.001. HM = hedonic motivation; SM = social motivation; KSM = knowledge-seeking motivation; CI = TCM cultural identity; HSE = health self-efficacy; HSD = TCM health care system distrust; HSI = TCM treatment intention.

Source(s): Authors’ own work.

### 4.5 The moderating effect of HSD

**Figure 2:**
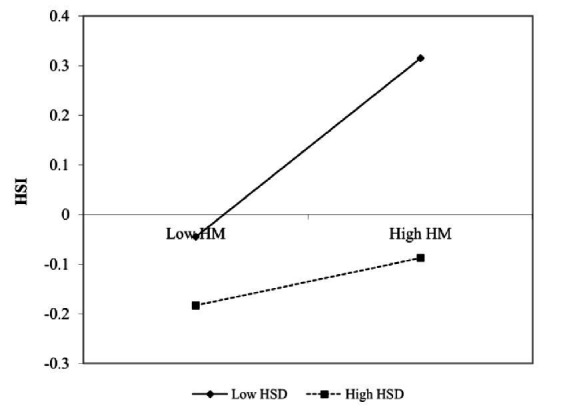
Moderating effect of HSD on the HM–HSI relationship.

**Figure 3:**
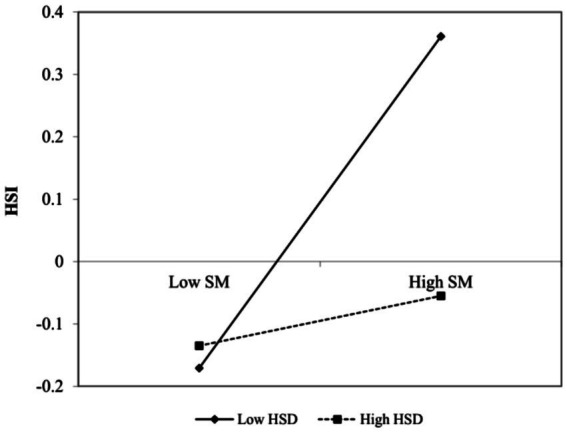
Moderating effect of HSD on the SM–HSI relationship.

**Figure 4:**
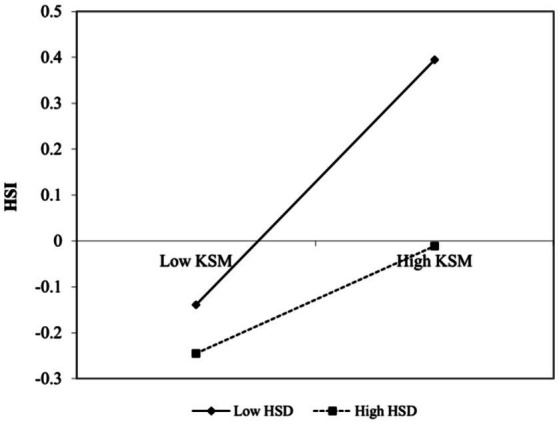
Moderating effect of HSD on the KSM–HSI relationship.

This adds calculated interaction indicators in the model based on Chin et al. (49). Table 8 illustrates the moderating effect of HSD on the relationship between gaming motivations and HSI. The results suggest that HSD exerts a negative moderating effect in the relationships between HM, SM, KSM and HSI, with SM demonstrating the largest negative mediating effect (β = −0.113, *p* < 0.05), while HM (β = −0.066, *p* < 0.05), and KSM (β = −0.075, *p* < 0.05) showed relatively weaker negative impacts. Thus, H6a, H6b, and H6c are all supported. Additionally, we generated simple slope plots for the three moderating effects. Figures 2–4 illustrate that HSD negatively moderates the relationships between HM, SM, KSM and HSI.

**Table 8:** The results of the moderating effect.

Moderator variable	Interacting	Dependent variable	β	*P*
HSD	HSD × HM	HSI	−0.066*	0.036
HSD	HSD × SM	HSI	−0.113*	0.036
HSD	HSD × KSM	HSI	−0.075*	0.022

*p < 0.05, **p < 0.01, ***p < 0.001. HM = hedonic motivation; SM = social motivation; KSM = knowledge-seeking motivation; CI = TCM cultural identity; HSE = health self-efficacy; HSD = TCM health care system distrust; HSI = TCM treatment intention.

Source(s): Authors’ own work.

### 4.6 Predictive relevance

The cross-validation redundancy and determination coefficients are the main indicators for evaluating the predictive relevance (44). The overall predictive power of the model is determined by the threshold values of R2. The R2 of TCM HSI was 0.722, which was greater than 0.6, representing larger predictive power (41), suggesting a large impact size. The R2 of HSE and CI were 0.437 and 0.386, demonstrating a medium effect size.

The relative predictive relevance of a model is primarily tested using the Stone-Geisser criterion. The Q2 of HSE, CI, and TMC HSI were 0.428, 0.374, and 0.633, which were all exceeding 0.35, and all exhibited large predictive relevance (50). Therefore, the results for R2 and Q2 demonstrated that the proposed model possesses strong explanatory and predictive relevance, validating its suitability for predicting HSI in the context of Chinese digital games. The results for R2 and Q2 are presented in Table 9.

**Table 9:** Fit indices for the model in the study.

Endogenous latent constructs	*R* ^2^	*Q* ^2^
HSE	0.437	0.428
CI	0.386	0.374
HSI	0.722	0.633

*p < 0.05, **p < 0.01, ***p < 0.001. CI = TCM cultural identity; HSE = health self-efficacy; HSD = TCM health care system distrust; HSI = TCM treatment intention.

Source(s): Authors’ own work.

## 5 Discussion

This study examined the impacts of different game motivations on intention to use TCM. The specific findings are as follows:

First, hedonic motivation, social motivation, and knowledge-seeking motivation serve as significant predictors of HSI but exert different impacts. Specifically, the total effects of knowledge-seeking motivation are stronger than those of hedonic motivation and social motivation. This result aligns with Zhang et al. (51), who reveal that health knowledge motivation serves as a proactive driver of treatment intention. While hedonic motivation shows significant paths, its direct effect on HSI is the lowest among the three. The present result diverges from Van der Heijden (52), who proved that in the use of an entertainment information system, the enjoyment is a stronger determinant than perceived usefulness. This may be because the actual pursuit of TCM treatment is inherently functional and risk-sensitive. Whereas digital games containing TCM cultural elements are hedonic in their delivery format, the target behavior shifts from gaming to seeking TCM treatment. Health-seeking behaviors are fundamentally rooted in an individual’s cognitive proficiency and informational certainty. Consequently, the sensory pleasure derived from game esthetics may attract initial interest, but it provides insufficient motivation to drive a serious healthcare intention. Therefore, players appear to adopt a utilitarian cognitive mode when evaluating the real-world value of TCM treatment. In this context, the pleasure of gaming serves as a secondary stimulus, while the knowledge-seeking motivation becomes the primary engine for behavioral intention.

Second, TCM cultural identification and health self-efficacy can influence the relationships between the three motivations and HSI. Specifically, health self-efficacy has a most pronounced mediating effect on the knowledge-seeking motivation and HSI. The finding is in line with the viewpoint of Social Cognitive Theory, which refers to the health belief in one’s capability to promote health management (53). It mediates the seeking of knowledge and treatment intention, regardless of whether the treatment is Western medicine or traditional Chinese medicine (54). Interestingly, the direct influence of Hedonic Motivation on HSI is relatively modest, its significant indirect effect through health self-efficacy. The plot and the characters are core factors of the enjoyment of digital games, when combined with players’ health self-efficacy, which can effectively drive players to shift from merely playing non-serious or commercial games to an enhancement toward traditional Chinese medicine treatment. Moreover, cultural identification of Chinese traditional medicine is a vital emotional mediator, particularly within the pathway driven by social motivation. This proves that social interaction in digital spaces can significantly foster cultural identity by providing a platform for shared values and group belonging. In the context of digital games containing TCM culture, when players engage in collective discussions or cooperative tasks, the culture identification can foster treatment intentions. This differentiated mediation pattern—wherein health self-efficacy more strongly mediates hedonic and knowledge-seeking motivations, while cultural identification more strongly mediates social motivation—can be explained by the core theoretical distinctions between social cognitive theory and social identity theory. Social cognitive theory, centered on individual cognition, identifies self-efficacy as the primary driver of behavioral intention (55). The individually oriented nature of hedonic and knowledge-seeking motivations, focused, respectively, on emotional experience and knowledge acquisition, allows in-game TCM elements to boost health self-efficacy and subsequent treatment intention, aligning with the theory’s individual-driven logic. In contrast, social identity theory emphasizes group belonging, framing cultural identity as the key behavioral driver in group settings (56). The group-oriented nature of social motivation, centered on peer interaction and identity affiliation, enables TCM elements to function as shared cultural symbols that strengthen cultural identification and drive treatment intention, fitting the theory’s group-driven logic.

Third, HSD functions as a negative moderating variable in the associations between hedonic motivation, social motivation, knowledge-seeking motivation, and TCM treatment intention. From the perspective of the theory of planned behavior, individuals’ trust in the health care system is a critical prerequisite for their acceptance of medical services, particularly in the context of non-Western medical systems or minority health-seeking groups (9). Even when digital games containing TCM culture activate individuals’ motivations, they may exhibit resistant behaviors and reduce their TCM treatment intention due to distrust in the TCM health care system. In this study, the negative impact of TCM health care system distrust on social motivation was the most pronounced. This is probably because players are more inclined to view TCM-related knowledge discussions within the gaming community as conversations about the game itself, rather than as professional and valid medical advice. In addition, the negative moderation of HSD on the relationship between hedonic motivation and TCM treatment intention was the mildest. This may be attributed to the fact that the other two motivations are both influenced by individuals’ rational value judgments, whereas hedonic motivation exerts a more affect-driven influence on players, resulting in the weakest negative moderation effect.

## 6 Conclusions, implications, and limitations

### 6.1 Conclusion

First, game motivations among players serve as crucial determinants of HSI, with differing magnitudes of influence. The total effect of knowledge-seeking motivation was more pronounced compared with other motivations. Second, hedonic motivation, social motivation, and knowledge-seeking motivation can influence HSI through TCM cultural identification and health self-efficacy, although the specific indirect effects are different. Health self-efficacy is a greater mediator of hedonic motivation-HSI and knowledge-seeking motivation-HSI than TCM cultural identity, while TCM cultural identity exerts a greater mediating effect between social motivation and HSI than health self-efficacy. Finally, the distrust of the TCM health care system moderates the indirect effect of the three motivation-HSI paths, whereas the moderating roles in the direct effect are not significant.

### 6.2 Implications

This study offers practical implications for Chinese game developers, the TCM health care system, and governments.

First, since knowledge-seeking motivation is the strongest determinant of HSI and health self-efficacy is a vital mediator, developers should move beyond purely esthetic portrayals of TCM. Game designers should regard TCM culture as an edutainment tool, rather than merely as part of the game background lore. Quest loops should encourage players to foster authentic TCM knowledge, such as herbal properties or meridian points, in order to solve in-game problems, as this builds the knowledge-seeking motivation that most effectively drives treatment intention. Because health self-efficacy is a more powerful mediator for learning-based paths than cultural identity, games should provide successful feedback when players complete health-related tasks. For example, when players cure NPCs or teammates by TCM treatment, games provide players with substantial rewards in order to boost their confidence in their own health management.

Second, for the TCM Healthcare System, the negative moderation of distrust suggests that they should improve their own medical technologies, take the initiative to disclose TCM-related medical information to the public, and enhance public trust. TCM hospitals and clinics must ensure the quality and transparency of real-world TCM services. Furthermore, TCM healthcare institutions should collaborate with Chinese game developers to embed trust within games. Specifically, they can implement in-game expert certification for all TCM content, integrate official health information modules linking to real hospital resources, and create virtual clinic experiences featuring actual physicians. This addresses the distrust barrier by reassuring players that the information they are learning is sanctioned by professional organizations. Clinics could use game-like digital interfaces or apps to provide patients with interactive health care, which may suspend their distrust during gameplay.

Third, governments play a crucial role in ensuring that the fun of gameplay leads to safe and effective public health outcomes. Policymakers should provide grants for those digital games that can balance hedonic motivation with knowledge-seeking motivation. Regarding those games as digital ambassadors for TCM helps foster players’ cultural identification. Governments must strengthen the regulation of the TCM healthcare market and improve medical insurance coverage for TCM to reduce the institutional distrust that currently throttles the conversion of digital interest into actual healthcare usage. Since knowledge-seeking motivation is the primary driver of TCM treatment intention, governments should integrate TCM-themed digital games into existing public health literacy frameworks—such as China’s *Healthy China 2030* and Digital Health strategic guidelines—as a supplementary tool to reach demographics resistant to traditional health education. These games should prioritize interactive knowledge application, embedding structured challenges that require players to learn authentic TCM content to progress, thereby enabling digital health campaigns to actively engage hard-to-reach populations.

### 6.3 Limitations

This study had three limitations. First, we did not distinguish the impacts of different types of diseases on TCM treatment intention, such as sub-health, acute illnesses, and major diseases.

Second, this study only explored the influence of the functional value. This may lead to an overestimation of HSI, as patients often bifurcate their trust between TCM and Western medicine based on the severity of the illness. In addition, the data were collected through an online questionnaire survey adopting self-reported measures, potentially resulting in measurement errors and sample selection bias. Actual behavioral data or experimental methods may be used in future studies. Finally, given that this study adopted a Chinese sample, the findings may not be generalizable to other cultural contexts.

## Data Availability

The raw data supporting the conclusions of this article will be made available by the authors, without undue reservation.

## Footnotes

1
https://www.wjx.cn/
