# Supplementary material for: Perceived enjoyment from virtual experience to real-world care: how Chinese digital games containing TCM culture influence players’ TCM treatment intention
Source: Front Public Health. 2026 Jun 2;14:1827198. doi: 10.3389/fpubh.2026.1827198 (PMC13269273; doi:10.3389/fpubh.2026.1827198)
Supplement: Supplementary file 1 [file Supplementary_file_1.docx]

**Supplementary Material: Sample Characteristics, Measurement Tests, and Analysis Results**

Table 1. Demographic profile of respondents

| Characteristic | Demographic | Frequency | Percentage |
| --- | --- | --- | --- |
| Gender | Male | 251 | 54.68% |
|  | Female | 209 | 45.32% |
| Age(years) | 20 and below | 8 | 1.74% |
|  | 21-30 | 148 | 32.24% |
|  | 31-40 | 246 | 53.38% |
|  | 41-50 | 40 | 8.71% |
|  | 51 and above | 18 | 3.92% |
| Educational | Senior high school or below | 7 | 1.53% |
|  | Associate degree | 36 | 7.84% |
|  | Bachelor degree | 368 | 80.17% |
|  | Master degree | 44 | 9.37% |
|  | Doctor degree | 5 | 1.09% |
| Marital status | Divorced/Widowed | 3 | 0.65% |
|  | Married | 357 | 77.78% |
|  | Unmarried | 100 | 21.57% |
| Gaming time(daily) | 1 hour per day | 123 | 26.80% |
|  | 2-3 hours per day | 297 | 64.49% |
|  | More than 4 hours per day | 40 | 8.71% |
| Gaming experience(years) | 1 year and below | 6 | 1.31% |
|  | 3-5 years | 97 | 21.13% |
|  | 6-7 years | 137 | 29.85% |
|  | 8 years and above | 220 | 47.71% |
| Total |  | 460 | 100 |

Source(s): Authors’ own work.

Table 2. Reliability and validity tests of the constructs

| Construct | VIF | Items | Standard loadings | Cronbach’s α | CR | AVE |
| --- | --- | --- | --- | --- | --- | --- |
| HM | 2.311 | Using Chinese digital games containing TCM culture is more fun than I initially expected | 0.892 | 0.849 | 0.908 | 0.768 |
|  | 1.884 | Actually process of using Chinese digital games containing TCM culture is more pleasant than I initially expected | 0.861 |  |  |  |
|  | 2.111 | Learning TCM knowledge by Chinese digital games containing TCM culture is more enjoyable than I initially expected | 0.876 |  |  |  |
| SM | 1.664 | I often have conversations with other players about TCM while playing games | 0.825 | 0.805 | 0.882 | 0.713 |
|  | 1.719 | It’s important for me to discuss with other players about TCM during the game | 0.843 |  |  |  |
|  | 1.745 | I really cherish the chance to talk about TCM with other players while playing games | 0.865 |  |  |  |
| KSM | 1.901 | Playing digital games containing TCM culture promotes my TCM knowledge growth and development | 0.861 | 0.824 | 0.895 | 0.739 |
|  | 1.807 | Playing digital games containing TCM culture reinforces my TCM competence | 0.856 |  |  |  |
|  | 1.867 | Playing digital games containing TCM culture helps me strengthen my concepts in TCM | 0.863 |  |  |  |
| CI | 1.57 | I have a strong sense of identification with the TCM culture presented in the game | 0.837 | 0.753 | 0.858 | 0.668 |
|  | 1.436 | I have an inexplicable sense of intimacy with the TCM culture presented in the game | 0.791 |  |  |  |
|  | 1.53 | I have a strong sense of belonging to the TCM culture presented in the game | 0.823 |  |  |  |
| HSE | 1.678 | I am confident that I could deal efficiently with my own health | 0.846 | 0.788 | 0.875 | 0.701 |
|  | 1.635 | I have the ability to deal with my health | 0.826 |  |  |  |
|  | 1.621 | When I am confronted with a health problem, I can usually find several solutions | 0.838 |  |  |  |
| TCM HDS | 1.51 | I believe that the TCM health care system puts making money above patients’ needs | 0.827 | 0.751 | 0.857 | 0.667 |
|  | 1.531 | I believe that the TCM health care system lies to patients for profit | 0.821 |  |  |  |
|  | 1.466 | I believe that the TCM health care system often leads to medical malpractice | 0.802 |  |  |  |
| HSI | 2.056 | I want to seek the TCM treatment if I encounter health problems | 0.882 | 0.821 | 0.893 | 0.736 |
|  | 1.758 | When I go to see a doctor, I will plan to actively consider TCM treatment | 0.845 |  |  |  |
|  | 1.787 | I intend to use TCM for health preservation and body regulation as part of my future health routine | 0.846 |  |  |  |

Source(s): Authors’ own work.

Table 3. Correlations and square roots of AVEs (Fornell-Larcker criterion)

|  | HM | HSE | KSM | SM | CI | HSD | HSI |
| --- | --- | --- | --- | --- | --- | --- | --- |
| HM | 0.876 |  |  |  |  |  |  |
| HSE | 0.573 | 0.837 |  |  |  |  |  |
| KSM | 0.647 | 0.585 | 0.860 |  |  |  |  |
| SM | 0.572 | 0.534 | 0.565 | 0.844 |  |  |  |
| CI | 0.505 | 0.604 | 0.532 | 0.546 | 0.817 |  |  |
| HSD | 0.432 | 0.542 | 0.447 | 0.542 | 0.578 | 0.816 |  |
| HSI | 0.641 | 0.716 | 0.681 | 0.650 | 0.688 | 0.632 | 0.858 |

Source(s): Authors’ own work.

Table 4. Heterotrait-Monotrait ratio (HTMT) and confidence interval

|  | HM | SM | KSM | CI | HSE | HSD |
| --- | --- | --- | --- | --- | --- | --- |
| HM |  |  |  |  |  |  |
| SM | 0.691 |  |  |  |  |  |
| KSM | 0.774 | 0.696 |  |  |  |  |
| CI | 0.632 | 0.702 | 0.676 |  |  |  |
| HSE | 0.702 | 0.670 | 0.725 | 0.783 |  |  |
| HSD | 0.542 | 0.699 | 0.567 | 0.766 | 0.703 |  |
| HSI | 0.768 | 0.799 | 0.828 | 0.876 | 0.890 | 0.806 |

Source(s): Authors’ own work.

Table 5 The results of the direct effect

| Hypotheses and paths | *β* | T-Vaule | Confidence intervals |
| --- | --- | --- | --- |
| HM -> HSE | 0.256*** | 5.096 | [0.156,0.349] |
| HM -> CI | 0.167** | 3.173 | [0.061,0.265] |
| HM -> HSI | 0.118** | 3.175 | [0.043,0.190] |
| HSE -> HSI | 0.244*** | 4.556 | [0.152,0.363] |
| KSM -> HSE | 0.294*** | 5.936 | [0.197,0.391] |
| KSM -> CI | 0.249*** | 4.596 | [0.143,0.356] |
| KSM -> HSI | 0.203*** | 5.491 | [0.130,0.275] |
| SM -> HSE | 0.222*** | 4.919 | [0.136,0.315] |
| SM -> CI | 0.309*** | 5.982 | [0.208,0.412] |
| SM -> HSI | 0.136** | 2.732 | [0.035,0.230] |
| CI -> HSI | 0.202*** | 3.812 | [0.098,0.303] |

Note(s): *p<0.05, **p<0.01, ***p<0.001

Source(s): Authors’ own work.

Table 6. The results of the mediating effect

| Hypotheses and paths | Specific indirect effects | | | Total indirect effects | | | Direct effects | | | Total effects | | |
| --- | --- | --- | --- | --- | --- | --- | --- | --- | --- | --- | --- | --- |
|  | *β* | T-value | Confidence intervals | *β* | T-value | Confidence intervals | *β* | T-value | Confidence intervals | *β* | T-value | Confidence intervals |
| HM -> CI -> HSI | 0.034* | 2.450 | [0.010,0.063] | 0.096*** | 3.986 | [0.053,0.147] | 0.118** | 3.175 | [0.043,0.190] | 0.214*** | 5.474 | [0.137,0.289] |
| HM -> HSE -> HSI | 0.062** | 3.081 | [0.029,0.109] |  |  |  |  |  |  |  |  |  |
| SM -> CI -> HSI | 0.062** | 2.850 | [0.025,0.109] | 0.117*** | 4.380 | [0.070,0.174] | 0.136** | 2.732 | [0.035,0.230] | 0.253*** | 5.714 | [0.164,0.337] |
| SM -> HSE -> HSI | 0.054** | 2.871 | [0.025,0.099] |  |  |  |  |  |  |  |  |  |
| KSM -> CI -> HSI | 0.050** | 2.835 | [0.020,0.089] | 0.122*** | 4.890 | [0.077,0.176] | 0.203*** | 5.491 | [0.130,0.275] | 0.325*** | 8.220 | [0.248,0.404] |
| KSM -> HSE -> HSI | 0.071*** | 3.504 | [0.038,0.119] |  |  |  |  |  |  |  |  |  |

Note(s): *p<0.05, **p<0.01, ***p<0.001

Source(s): Authors’ own work.

Table 7. The results of the moderating effect

| Moderator  variable | Interacting | Dependent variable | *β* | *p* | Support |
| --- | --- | --- | --- | --- | --- |
| HSD | HSD x HM | HSI | -0.066* | 0.036 |  |
| HSD | HSD x SM | HSI | -0.113* | 0.036 |  |
| HSD | HSD x KSM | HSI | -0.075* | 0.022 |  |

Note(s): *p<0.05, **p<0.01, ***p<0.001

Source(s): Authors’ own work.

Table 8. Fit indices for the model in the study

| Endogenous latent constructs | *R^2^* | *Q^2^* |
| --- | --- | --- |
| HSE | 0.437 | 0.428 |
| CI | 0.386 | 0.374 |
| HSI | 0.722 | 0.633 |

Source(s): Authors’ own work.
